# Upcycling of By-Products from Autochthonous Red Grapes and Commercial Apples as Ingredients in Baked Goods: A Comprehensive Study from Processing to Consumer Consumption

**DOI:** 10.3390/antiox14070798

**Published:** 2025-06-27

**Authors:** Gaetano Cardone, Martina Magni, Veronica Marin, Andrea Pichler, Daniele Zatelli, Peter Robatscher, Ombretta Polenghi, Virna Lucia Cerne, Michael Oberhuber, Silvano Ciani

**Affiliations:** 1 Dr. Schär Research & Development Department, 34139 Trieste, Italy; gaetano.cardone@drschaer.com (G.C.); veronica.marin@drschaer.com (V.M.); ombretta.polenghi@drschaer.com (O.P.); virna.cerne@drschaer.com (V.L.C.); 2 Laboratory of Flavours and Metabolites, Laimburg Research Centre, Laimburg 6, 39040 Auer-Ora, Italyandrea.pichler@laimburg.it (A.P.); michael.oberhuber@laimburg.it (M.O.); 3 VOG Products, Soc. Agricola Coop, 39055 Laives, Italy; daniele.zatelli@vog-products.it

**Keywords:** polyphenols, grape pomace, apple skin, UHPLC–MS/MS, gluten-free, circular economy

## Abstract

Lagrein grape (*Vitis vinifera* L.) pomace and Scilate apple (*Malus domestica* Borkh.) skin are polyphenol- and antioxidant-rich by-products with promising applications in the food industry. This study investigated the impact of drying and grinding on their antioxidant properties for use in gluten-free baked goods. Regardless of the by-product analysis, the results showed that processing conditions effectively preserved most of the polyphenols. Furthermore, the grape pomace and apple skin flours produced retained approximately 86% and 66% of anthocyanins, respectively. Incorporating these flours into breadsticks, focaccia, and cookies significantly enhanced their polyphenol content (300–727%), anthocyanin content (600–1718%), and antioxidant capacity (280–1200%). The addition of these by-products to baked goods led to a slight decrease in texture and sensory properties. However, adding both grape pomace and apple skin flours significantly improved consumer acceptance compared to products containing only grape pomace flour. This study highlights the potential of upcycling by-products from grapes and apples to enhance the nutritional profile of gluten-free products while supporting a circular economy approach.

## 1. Introduction

South Tyrol, Italy, is an important agricultural region, particularly known for the production of wine and apples. Grapes are the third most important agricultural commodity in Italy after milk and maize, with an annual wine production of about 5 million tons [1]. Among the autochthonous red grape cultivars, *Lagrein cv* is notable for its high content of phenolic compounds [2]. Similarly, apple cultivation plays a key role in South Tyrol’s economy, with an average harvest of 950,000 tons per year, accounting for approximately 50% of Italy’s apple production [3]. Roughly 11% of these apples are processed into juices, concentrates, and purees, resulting in significant amounts of both by-products—primarily grape pomace and apple peels [4]. Despite being used for alcohol distillation (grape pomace) and commonly discarded or used as animal feed (apple peels), these materials are still rich in valuable nutrients and bioactivates such as polyphenols and antioxidants [5,6,7]. Studies have identified three major classes of bioactive compounds in grape skins: phenolic acids, stilbenes, and flavonoids, including flavan-3-ols and conjugated anthocyanins in black grape varieties. Grape seeds are also rich in fibers, proteins, fats, and flavan-3-ols [2]. Similarly, apple by-products contain phytochemicals such as carotenoids, flavonoids, and phenolic acids, including chlorogenic acid, phloridzin, epicatechin, procyanidin B2, and quercetin [7], with strong antioxidant properties. Their concentration is influenced by multiple factors, such as fruit variety, geographical origin, cultivation period, ripeness, and post-harvest storage conditions.

Polyphenols and related compounds are widely studied for their antioxidant, anti-inflammatory, and anticancer properties, and have been associated with the prevention of chronic conditions such as cardiovascular diseases and diabetes [8,9]. Moreover, recent findings suggest that dietary polyphenols can positively influence gut microbiota composition, contributing to improved metabolic and immune function [10,11,12].

From a sustainability standpoint, the reuse of fruit by-products aligns with circular economy principles and represents a promising strategy to improve the nutritional profile of foods while reducing food waste. This is particularly relevant for gluten-free (GF) products, which are often characterized by lower fiber and antioxidant contents compared to their wheat-based counterparts [13]. However, the incorporation of antioxidant-rich by-products into baked goods presents technological challenges. Indeed, thermal processes such as drying and baking can lead to the degradation of heat-sensitive compounds [14,15,16].

To preserve antioxidant functionality throughout processing, it is essential to adopt optimized technologies such as low-temperature drying and cryogenic grinding. Several studies have examined the enrichment of conventional baked goods with grape or apple by-products [17,18,19], yet few have focused on their application in GF matrices [20,21], where technological constraints and consumer expectations (e.g., appealing and acceptance) differ significantly [20].

Our goal was to incorporate antioxidant-rich matrices derived from by-products into three types of gluten-free baked goods (i.e., breadsticks, focaccia, and cookies) at levels that would maximize polyphenol and antioxidant content in the final products while minimizing the impact on the final product’s technological properties. Regardless of the product types, grape pomace flour was incorporated at a 5% level, while apple skin flour was added at a 3% level in savory products (focaccia and breadsticks) and 10% in sweet ones (cookies). The study evaluates the polyphenol profile, anthocyanin content, and antioxidant capacity of fresh, dried, and ground by-products from *Lagrein grape* and *Scilate apple*. Moreover, the antioxidant profile, technological properties (hardness, friability, and color) and consumer acceptability of gluten-free baked goods, comparing unenriched and enriched formulations, were also investigated. This comprehensive approach seeks to demonstrate the potential of upcycling food by-products into value-added, health-promoting gluten-free baked goods while addressing key processing and sensory challenges.

## 2. Materials and Methods

### 2.1. Chemical and Reagents

All the reagents and standards used for this work were at least analytical grade and ultrapure water was used (Integral 15, Millipore, Burlington, MA, USA).

Astilbin, chlorogenic acid, cryptochlorogenic acid, neochlorogenic acid, cyanidin-3-arabinoside, gallocatechin, isorhamnetin-3-rutinoside, malvin, malvidin-3,5-diglucoside, phloretin, petunidin, petunidin-3-glucoside, procyanidin B1, procyanidin B2, quercetin-3-glucuronide, quercetin-4′-O-glucoside, syringic acid and taxifolin were purchased from Phytolab GmbH & Co. (Vestenbergsgreuth, Germany); (+)-catechin, delphinidin-3-glucoside, kaempferol, gallic acid from Carl Roth (Karlsruhe, Germany); (±)-Catechin-2,3,4-^13^C_3_, myricetin, quercetin-3-glucoside, quercetin-3,4′-diglucoside, quercetin-3-rutenoside, quercetin-3-arabinoside, phloridzin dihydrate, phosphoric acid, prunin, procyanidin C1, quercetin-3-rhamnoside, quercetin-3-xyloside, anhydrous sodium carbonate, acetic acid (100%), potassium chloride (99.5%), sodium fluoride (≥99.9%), Folin–Ciocalteu’s phenol reagent (Product Code: 101655216), “37 component FAME mix standard”, heptane (GC-FID grade) from Sigma Merck KGaA (Darmstadt, Germany); cyanidin-3-glucoside from ChromaDex (Los Angeles, CA, USA); cyanidin-3-galactoside, epigallocatechin, kaempferol-3-glucoside, kaempferol-3-glucuronide, kaempferol-3-rutinoside, isorhamnetin, isorhamnetin-3-glucoside, myricetin-3-glucoside, quercetin, quercetin-3-galactoside from Extrasynthese (Genay, France); (-)-epicatechin from Applichem (Darmstadt, Germany); ferric chloride hexahydrate (≥99%), 2,4,6-Tris(2-pyridyl)-s-triazine or TPTZ (CAS: 3682-35-7), anhydrous sodium acetate (99.9%) from Fisher Scientific (Segrate, Italy); formic acid (LC-MS grade) from Chem-Lab Analytical (Zedelgem, Belgium); potassium hydroxide (≥85%) from Carlo Erba reagents (Cornaredo, IT); anhydrous sodium sulphate (>98.5%) from Titolchimica (Pontecchio Polesine, Italy) and trans-caftaric acid, acetonitrile (LC-MS grade), methanol (UHPLC-MS grade), hydrochloric acid (fuming, ≥37%) from Honeywell/Fluka (Cologno Monzese, Italy).

### 2.2. Flours from Grape Pomace and Apple Skin

**Figure 1:**
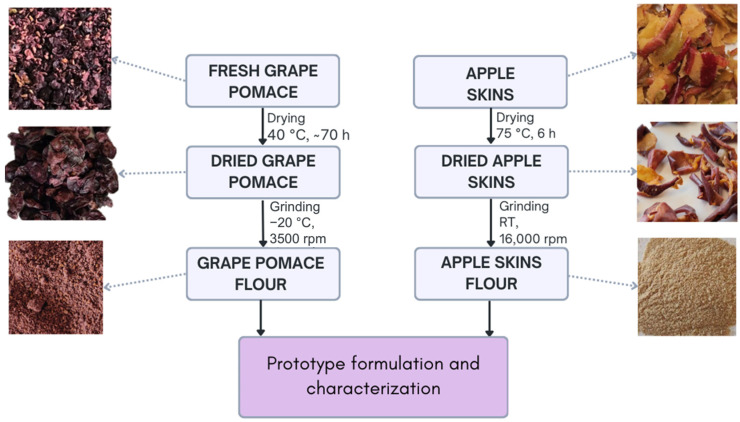
Grape pomace and apple skin flour production. (RT: room temperature).

Red grape pomace (skin and seeds) and apple skin used in this study (Figure 1) were obtained from the *Lagrein cv* (*Vitis vinifera* L.) and *Scilate cv* (*Malus domestica* Borkh.), both harvested in 2022 in South Tyrol, Italy. Once the grapes were fermented, the pomace was recovered and dried at 40 ± 2 °C for about 70 h until 11% moisture was reached. Apple skins were recovered immediately after the peeling step and dried at 75 ± 2 °C for 6 h until 2.5% moisture.

An aliquot of dried grape pomace was ground at room temperature using a mixer mill (MM 400 Retsch, Verder Scientific GmbH & Co. K.G., Golling, Austria) to obtain a sample, referred to as “dried grape pomace”, for evaluating the effects of the drying step. A second aliquot was cryogenically ground using a CRIO-Contraplex 250 CWII mill (Hosokawa Alpine AG, Augsburg, Germany) at −20 °C and 3500 rpm, producing grape pomace flour with 90% of particles under 250 µm. This flour, referred to as “grape pomace flour”, was used to assess the combined effects of both drying and grinding processes and it was used as raw material in baked goods. A part of dried apple skin was ground at 6000 rpm using a mixer mill, obtaining a flour, referred to as “dried apple skin”, used to evaluate the effects of drying. Another part was ground at 16,000 rpm with an ultra-centrifugal mill (ZM 200 Retsch, Verder Scientific GmbH & Co. K.G.) obtaining a flour (particle size < 500 μm), referred to as “apple skin flour”, used to assess the combined effects of both drying and grinding processes and it was used as a raw material in baked goods. The selected drying and milling conditions were based on preliminary internal trials aimed at maximizing antioxidant retention. Specifically, drying temperature and duration, as well as milling speed and temperature, were optimized to minimize thermal and oxidative degradation of polyphenols and anthocyanins and ensure suitable particle size for sensory acceptability. Attention to these parameters was essential due to the presence of seeds in the grape pomace, which require efficient milling to prevent the formation of coarse particles noticeable during mastication. Apple skins, being softer and seed-free, were ground to <500 mm without affecting texture or mouthfeel. These choices ensured both ingredient functionality and sensory acceptability.

### 2.3. Chemical Characterization of By-Products and Baked Goods

#### 2.3.1. Sample Preparation for Analysis

Samples of fresh by-products and baked goods were freeze-dried before analysis (−50 °C, 0.080 mbar, 5 days) and then ground with a mixer mill to homogenize them. The dried grape pomace, grape pomace flour, dried apple skin, and apple skin flour obtained under the conditions described in the Section 2.2 were used without further processing.

#### 2.3.2. Extraction of Samples

The extraction was carried out according to the procedure described in the book *Food Analysis* [22]. In a 2 mL Eppendorf tube, 25 mg of powdered sample, 1800 µL of solution A (phosphoric acid 100 mM in methanol and water 80:20 (*v*/*v*)) and 30 µL of solution B (sodium fluoride 100 mM) were added. Then, the tubes were shaken in a thermomixer (Thermomixer comfort, Eppendorf, Hamburg, Germany) at 1400 rpm for 15 min at 5 °C. A second extraction step was performed on the by-product flours, by removing the supernatant solution and adding 1800 µL of solution A and 30 µL of solution B; after shaking and centrifugation, the two extraction phases were combined. Centrifugation (Centrifuge 5810 R, Eppendorf, Hamburg, Germany) is performed as the final step at 20,000× *g* for 5 min at 5 °C.

#### 2.3.3. Spectrophotometric Assays of Fresh and Dried By-Products

##### Total Polyphenol Content: TPC

Following Wolfe et al. [23], the Folin–Ciocalteu assay was performed in order to estimate the TPC of the samples: 60 µL of the sample extract was put into a 2 mL Eppendorf tube with 250 µL of water and 60 µL of the Folin–Ciocalteu solution (diluted 1:4 (*v*/*v*) with water), mixed at 1400 rpm for 6 min at 20 °C in the thermomixer, then 630 µL of sodium carbonate solution 7% (*w*/*v*) was added and shaken at 1400 rpm for 90 min at 20 °C. Afterwards, the absorbance was read at 740 nm on a UV–Vis spectrophotometer (Cary 60, Agilent Technologies, Santa Clara, CA, USA). The calibration curve was built using gallic acid from 0 to 500 mg/L. Analysis was carried out in duplicate and results are expressed as mg of gallic acid equivalents per 100 g dry weight (DW) of sample (mg_GAE_/100 g DW).

##### Total Anthocyanins: TA

Following Lee et al. [24], the TA content was determined by using an aliquot of 200 µL of sample extract and 800 µL of potassium chloride 0.25 M (pH = 1). After manual shaking, absorbance was read at 520 and 700 nm, and the total content of anthocyanins was calculated by the Lambert–Beer law. Analysis was carried out in duplicate and TA are expressed as mg of cyanidin-3-glucoside equivalents per 100 g DW of sample (mg_cya-3-glu eq_/100 g DW).

##### Ferric-Reducing Antioxidant Potential: FRAP

Following Benzie et al. [25], the FRAP assay was performed to estimate the samples’ antioxidant capacity. In a 2 mL Eppendorf tubes, 180 µL water, 60 µL of sample extract and 960 µL of FRAP reagent (acetate buffer 0.3 M, TPTZ solution 10 mM and FeCl_3_ 20 mM with proportion 10:1:1 (*v*/*v*)) was mixed for 90 min at 1400 rpm at 37 °C in the thermomixer. Afterwards, the absorbance was read at 595 nm. The calibration curve was built from 0 to 1 mM of Fe^2+^ in water. Analysis was carried out in duplicate, and results are expressed as mmol of ferrous ions per 100 g DW of sample (mmol Fe^2+^/100 g DW).

#### 2.3.4. Mass Spectrometric Analyses of Fresh and Dried By-Products

##### Single Polyphenol Compounds: Grape Pomace

Following Valls et al. [2], single polyphenolic compounds were analyzed with HPLC-MS for grape samples: 20 mg of freeze-dried, milled sample was weighed into a 15 mL Falcon tube, with 30 µL of ^13^C_3_-gallocatechin (100 mg/L) as surrogate standard and 6 mL of a 50:50 (*v*/*v*) solution of methanol and water + 1% formic acid. The tube was shaken with a vortex (20 s, 100 rpm, 25 °C ± 1 °C; vortex parameter: reciprocal 45°, 40 s; vibro 5°, 5 s; Multi-Rotator PTR-60, Grant Instruments, Royston, UK), left in an ultrasonic bath for 10 min at 25 °C ± 1 °C, then centrifuged (10 min, 3000× *g*, 5 °C). The supernatant was transferred to another falcon tube, and the extraction step was repeated for a second time. The second extract was mixed with the first one. In an LC glass vial, 1000 µL of the whole extract was mixed with 50 µL of quercetin-4′-O-glucoside, 5 mg/L, used as an Internal Standard (I.S.). The external calibration curve was established from 0.0025 to 10 mg/L. The I.S. was added into each vial of the calibration curve in the same way described for samples. Each sample was analyzed in duplicate; the average value with the relative standard deviation is reported.

Samples, calibration curve, blanks and solvent were analyzed by UltiMate 3000 UHPLC system (Thermo Scientific, Waltham, MA, USA) coupled with a triple quadrupole mass spectrometer TSQ Quantiva (Thermo Scientific, Waltham, MA, USA), equipped with an H-ESI source, where the parameters were set as follows: positive voltage 5000 V, negative voltage 3400 V, capillary temperature 325 °C, vaporizer temperature 320 °C, sheath gas 30 arbitrary unit (Arb), auxiliary gas 15 Arb, sweep gas 1 Arb. The collision gas (argon) was set at 1.5 mTorr. The chromatographic column Hypersil GOLD™ C18 (100 × 2.1, 1.9 µm, Thermo Scientific, Waltham, MA, USA), equipped with the relative pre-column, was used with water as solvent A and acetonitrile as solvent B, both acidified using 1% formic acid; the flow rate was 0.3 mL/min. The initial conditions were: 3% B held for 2 min, then until 18 min, reached 45% B, and in 1 min, up to 100% B for the washing step, returning to the initial conditions, and then the reconditioning step. The column compartment temperature was set to 30 °C; the autosampler to 12 °C. Data were processed using TraceFinder 3.2 (Thermo Scientific, Waltham, MA, USA). Complete information about *m*/*z* of the investigated fragments for each compound, collision energies, and other settings of the mass spectrometer are summarized in Supplementary Table S1. The quantification was made on each standard’s relative external calibration curve and is expressed as mg per 100 g DW of sample (mg/100 g DW).

##### Single Polyphenol Compounds: Apple Skins

Following Valls et al. [2], single polyphenolic compounds were analyzed by HPLC-MS for apple skins, as follows: 25 mg of freeze-dried, milled sample was weighed into a 2 mL Eppendorf tube, with 30 µL of quercetin-4′-O-glucoside (15 mg/L) as surrogate standard, 1800 µL of a 80% methanol solution acidified using phosphoric acid and 30 µL of sodium fluoride solution (100 mM). The tube was shaken with a thermomixer (15 min, 1400 rpm, 5 °C ± 1 °C), then centrifuged (10 min, 3000× *g*, 5 °C). For the injection vial preparation: 75 µL of the supernatant was transferred to a glass vial equipped with a glass insert, 75 µL of milliQ water was added, plus 7.5 µL of the Internal Standard (Malvidin-3-glucoside, 15 mg/L). The external calibration curve from 0.005 to 10 mg/L was prepared by diluting a mother mix solution containing all analytes. Each sample was analyzed in duplicate: the average value with the relative standard deviation is reported.

Samples, calibration curve, blanks and solvent were analyzed by UltiMate 3000 UHPLC system (Thermo Scientific, Waltham, MA, USA) coupled with a triple quadrupole mass spectrometer TSQ Quantiva (Thermo Scientific, Waltham, MA, USA), equipped with an H-ESI source, where the parameters were set as follows: positive voltage 1500 V, capillary temperature 325 °C, vaporizer temperature 320 °C, sheath gas 40 Arb, auxiliary gas 15 Arb, sweep gas 2 Arb. The collision gas (argon) was set at 1.5 mTorr. The chromatographic column Hypersil GOLD™ C18 (50 × 2.1, 1.9 µm, Thermo Scientific, Waltham, MA, USA), equipped with the relative pre-column, was used, with water as solvent A and acetonitrile as solvent B, both acidified using 2.5% formic acid, the flow rate was 0.5 mL/min. The initial conditions were 2.5% B, held for 1 min, then for 10 min, to 16.5% B, held for 1.5 min, in 1 min 23.5% B was reached, in 2.5 min 55% B was reached, and in 0.5 min 95% B was reached for the washing step, returning to the initial conditions, and then the conditioning step. The column compartment temperature was set to 40 °C and the autosampler to 12 °C. Data were processed using TraceFinder 3.2 (Thermo Scientific, Waltham, MA, USA). Complete information about *m*/*z* of the investigated fragments for each compound, collision energies, and other settings of the mass spectrometer are summarized in Supplementary Table S2. The quantification was made on each standard’s relative external calibration curve and is expressed as mg per 100 g DW of sample (mg/100 g DW).

#### 2.3.5. Total Fat Content: Grape Pomace

In accordance with the European Commission Directive 98/64/EC, Part B: “Determination of Oils and Fats” [26], 5 g of grape pomace flour was weighed into a Soxhlet extraction thimble, covered with a cotton plug, and placed in the extractor. Lipids were extracted for 6 h using petroleum ether as the solvent. The extract was subsequently collected in a flask and concentrated to dryness using a rotary evaporator (Büchi, Flawil, Switzerland).

#### 2.3.6. Fatty Acid Methyl Esters (FAME): Grape Pomace

Referring to the official standard method for FAME analysis ISO 12966-2-2017 [27] and quantification ISO 12966-4-2015 [28], the fat was extracted as follows: 30 g of freeze-dried, milled sample was weighed in a beaker and covered with heptane, mixed manually and filtered under vacuum; the solvent was dried using a rotary evaporator until no organic solvent trace remained; 0.2 g of fat was weighed into a screwed cap vial and dissolved in 2 mL of heptane; 2 mL potassium hydroxide (2 M in methanol) was added, the vial was closed and shaken vigorously for a few seconds. After phase separation, the organic phase was dried over sodium sulfate and transferred into a GC glass vial.

For the gas chromatographic determination, the 37-component FAME mix standard was used, GC-FID (GC 2030, Shimadzu, Kyoto, Japan) was equipped with SH-2560 Shimadzu column (105 m × 0.25 mm ID, 0.2 µm) using hydrogen as carrier gas, and the following temperature settings: 70 °C held for 1.2 min, with a rate of 8.3 °C/min to 225 °C, held for 5.4 min and with the same rate to 240 °C, held for 15 min. The injection volume was 1 µL. Analysis was carried out in a single repetition, and results are expressed as relative percentages (%) for each single peak area referred to 100% as the total peak area measured (Supplementary Table S3).

### 2.4. Baked Goods

Gluten-free breadsticks, focaccia, and cookies to which the grape pomace and apple skin flour were added, were produced at a lab scale at Dr. Schär R&D Centre (Dr. Schär SPA, Postal, Italy), kneaded using a Kenwood mixer (model KVC5300S, Havant, UK) and baked (electric oven MIWE Condo, MIWE GmbH, Arnstein, Germany). Table 1 reports the maximum amount allowable in the product formulation evaluated through preliminary tests. These levels were determined to ensure the dough exhibited appropriate handling, leavening, and structural characteristics. Enriched baked goods were compared with unenriched products without by-products to assess the post-baking recovery of the antioxidants in baked goods. Two baking tests were performed for baked goods production, and twenty breadsticks, three focaccia, and twenty cookies were produced from each baking test.

For the sensory characterization, in addition to the unenriched sample and the enriched sample containing both grape pomace and apple skins, an additional formulation containing only 5% grape pomace was also tested.

#### 2.4.1. Food System Breadsticks

Unenriched breadsticks were formulated as follows: potato starch, oats (wholegrain flour and fiber), oil and margarine, hydrocolloids, chicory fiber, fresh yeast, and salt. All the ingredients were kneaded for 10 min, the resulting dough was processed using an automatic sheeter to achieve 1.5 cm thickness, and the breadsticks were handmade. The leavening phase of the breadsticks took place for 55 min at 30 °C. Once the dough had leavened, the breadsticks were baked at 210 °C for 16 min.

#### 2.4.2. Food System Focaccia

Unenriched focaccia was formulated with corn starch, oat (wholegrain flour and fiber), hydrocolloids, chicory fiber, oil, fresh yeast, and salt. All these ingredients were kneaded for 13 min, and the resulting dough was rolled out to a thickness of 1.5 mm and cut to obtain portions of focaccia of 120 g. The leavening phase was conducted for 50 min at 30 °C. After leavening, the focaccia was baked at 190 °C for 18 min.

#### 2.4.3. Food System Cookies

Unenriched cookies were made using oats (flour, flakes, and fiber), margarine, chicory fiber, soluble fiber, baking powder, and citric acid. All the ingredients were kneaded for 10 min, and the resulting dough was hand-sheeted to achieve a thickness of 0.5 cm. The resulting cookies were baked at 170 °C for 21 min.

### 2.5. Technological Characterization of Baked Goods

#### 2.5.1. Color Analysis

The internal color of focaccia, breadsticks, and cookies were determined by a digital colorimeter (Digital Color Meter, Apple Inc., Cupertino, CA, USA), expressing results in CIE L*a*b* color space. The crumb and crust color assessment of focaccia and the outside color of breadsticks and cookies were replicated five times.

#### 2.5.2. Texture Profile Analysis

Focaccia was characterized by a Texture Profile Analyzer (TA.XT-plus, Stable Micro Systems Ltd., Godalming, UK), as previously reported by Perri et al. [29]. Samples were analyzed 2 h after baking and after 30, 60, and 90 days and stored in a plastic bag at 20 °C until the test. Instead, cookies and breadsticks were evaluated in terms of hardness and fracturability, according to Rainero et al. [30]. These samples were analyzed 2 h after baking and after 55 and 110 days of accelerated storage at 45 °C, simulating 9 and 12 months of storage under ambient conditions, respectively. Hardness and friability were carried out on ten breadsticks, cookies, and six focaccia.

### 2.6. Impact of Baking on Bioactive Compounds

The impact of baking on the stability of bioactive compounds was calculated by the theoretical contributions of the total polyphenols and anthocyanins expected from the added by-products and compared with the content measured in the baked goods. Thermal retention was estimated indirectly and calculated as follows:

$$Thermal\;retention\;\left(\%\right)=\frac{Bioactive\;compound\;in\;baked\;goods}{\sum \left(\%ingredient_{i}\cdot C_{i}\right)}\cdot 100$$

where C_i_ is the content of bioactive compounds (mg/100 g DW) in the grape pomace and apple skin flours, and %ingredient_i_ refers to the percentage of each by-product flour used in the formulation. Complete information about antioxidant profile retention are summarized in Supplementary Table S5.

### 2.7. Sensory Evaluation and Consumer Test

#### 2.7.1. Sensory Test

Nine panelists performed the sensory descriptive analysis. Specifically, the sensory attributes chosen for breadsticks and focaccia characterization were: sourish and fruity odor; salty, sour, and fermented taste; fruity and off flavor. Regarding cookies, the descriptors were: sourish and fruity odor; sweet, bitter, and fermented taste; fruity and off flavor. The panelists used a 9-point scale from “no perceived” (1, left side of the scale) to the “highest intensity perceivable” (9, right side of the scale) to describe samples.

#### 2.7.2. Consumer Test

The analysis was carried out at the Sensory Analysis and Consumer Science Laboratory (SCS_lab; according to ISO/DIS 8589:2007 [31]) of the DeFENS (Department of Food, Environmental, and Nutritional Sciences) of the University of Milan, Italy. Seventy-five participants (55% women and 45% men between 22 and 65 years old) were selected from the students and staff. The study followed the Declaration of Helsinki, and all participants had informed consent before participation.

The samples were stored at room temperature, away from light and heat sources, until tasting and were served to tasters on plastic plates or in cups coded with three-digit numbers, respectively. The samples were provided together with still water.

An acceptability/preference method (ISO FDIS 11136:2014 [32]) was carried out on the focaccia and breadstick samples. Consumers were asked to provide an overall rating of liking, using a linear scale anchored at the extremes of “extremely disliked” (corresponding to the 0 value on the left of the scale) to “extremely liked” (corresponding to the 100 values on the right of the scale). Next, consumers were asked to answer three questions regarding the compliance, for each sample, of the following sensory attributes: color, flavor, and texture in the mouth. Consumers were also asked to respond to a questionnaire concerning attitudes toward enriched products, sustainability, eco-circulation, and recovery of food industry by-products. The questionnaire consisted of seven statements for each of which respondents were asked to indicate the degree of agreement on a 5-point scale still with the extremes of “strongly disagree” (1, left side of the scale) to “strongly agree” (5, right side of the scale).

### 2.8. Statistical Analysis

The data are expressed as the arithmetic average ± standard deviation. One-way ANOVA (α = 0.05) and Tukey HSD test was carried out to establish differences between the three group samples. Then, a paired *t*-test was carried out to determine differences between two group samples. All data were elaborated by Minitab 22.1 (Minitab Inc., State College, PA, USA).

## 3. Results and Discussion

### 3.1. Stability of Bioactive Compounds During Drying and Milling

Preserving the stability of the bioactive compounds, known for their significant health benefits, is crucial for enhancing the nutritional quality of enriched foods.

The effects of the drying and grinding steps on the total polyphenol and anthocyanin contents, as well as the antioxidant capacity of grape pomace and apple skin, are presented in Table 2.

The polyphenol and anthocyanin contents of fresh grape pomace used in this study are consistent with existing literature, confirming the high content of antioxidants of *Lagrein cv* [2]. Negro et al. [33] reported a range of 2760 to 5290 mg_GAE_/100 g DW for total polyphenols in four red grape varieties, including Negroamaro, Malvasia di Lecce, Primitivo, and Cabernet *Sauvignon cvs*. Additionally, the total anthocyanin levels observed were consistent with those reported for *Terci cv* (~415 mg_Cya-3-glu eq_/100 g DW) by Ribeiro et al. [34], though lower anthocyanin contents were noted in Cabernet Sauvignon and *Merlot cvs*, at ~150 and ~76 mg_Cya-3-glu eq_/100 g DW, respectively. Regarding antioxidant capacity, our results (80 mmol Fe^2+^/100 g DW) were higher compared to approximately 2 mmol Fe^2+^/100 g DW reported by Ribeiro et al. [35] for *Merlot cv*. Such variability might be attributed to grape variety, environmental conditions, climate, agronomic practices and harvesting year.

After racking, red grape pomace still contains about 50% moisture, representing an optimal substrate for microorganisms, such as molds and yeasts [36]; for this reason, a drying process was applied to improve matrix stability during storage. It should be mentioned that, although fresh grape pomace is rich in bioactive compounds, polyphenols are thermolabile and can degrade during heat-based processing, such as drying [37,38]. In this study, a dryer plant with high loading capacity was used to obtain dryness without extended loss of antioxidants due to prolonged contact with oxygen in air at mild temperatures (40 °C). For the same reason, to prevent antioxidant degradation and gear jamming, due to the high lipid content of grape seeds, the grinding was carried out on a cryo-plant at low temperature (−20 °C). In general, our results showed that the functional properties of grape pomace were slightly affected by drying and grinding (Table 2). The reduced antioxidant profile in the dried grape pomace, as opposed to the grape pomace flour, could be attributed to the use of different grinding scales (laboratory vs. industrial). The industrial grinding resulted in a flour with smaller particle size compared to the laboratory method (see Supplementary Table S4), leading to a higher extraction efficiency of the analytes. Our results are consistent with Pramsohler et al. [39] who noted that a finely ground herb mixture showed a statistically different pesticide content, albeit minor, compared to the mixture with larger particle size. This difference is likely attributed to the small particles in the finely ground mixture having a larger surface area than the shredded herbs in the mixture with larger particles, making them more efficiently extracted by the solvent. On the other hand, Sokač et al. [40], observed that the polyphenol content of skins, seeds, and grape pomace, was not strongly affected by different drying methods (conventional, vacuum drying, and open sun drying) at different temperatures, ranging from 40 to 70 °C. Additionally, the greater preservation of polyphenols might also be due to the fact that the grape seeds were mostly enclosed in the grape skin, which protected them from thermal damage. Indeed, polyphenols contained in the intact seeds enclosed in grape skin are less prone to temperature-induced degradation compared to those present in potentially broken seeds during pressing [41]. Regarding apple skins, to the best of our knowledge, the available literature on the antioxidant profile of the Scilate cv is limited. However, our findings (Table 2) for fresh apple skin align with reported values for total polyphenols, anthocyanins, and antioxidant capacity in other red apple varieties, such as Jonagold and Royal Gala [42,43]. In any case, the antioxidant profile of apples is influenced by the cultivar. Unlike total polyphenols, the drying of apple skin led to a decrease by 34% of anthocyanins and consequently a decrease in the antioxidant capacity (by about 14%). This decrease may be attributed to the greater susceptibility of anthocyanins to thermal degradation than other polyphenols, such as proanthocyanidins or phenolic acids, which are more stable at moderate temperatures. Previous studies have shown that anthocyanin loss during thermal processing depends on both temperature and exposure time, as well as on interactions with other matrix components, such as sugars and organic acids, which may either stabilize or accelerate degradation [44,45]. Ma et al. [46] reported a more substantial decrease in polyphenol content (−38%) and antioxidant capacity (−42%) when red apple skins were dried at 75 °C using the hot air-drying method, than fresh samples. Heras-Ramírez [47] also reported a decrease in total polyphenol content of 75% and anthocyanin content of 100% in Red Delicious *cv* after drying at 70 °C.

### 3.2. Polyphenols and Anthocyanin Composition in Flours

Alongside the Folin–Ciocalteu assay, a specific quantification of single polyphenols using mass spectrometric detection (HPLC-MS) was conducted, to gain a deeper insight into the effect of the technological processes on these compounds. Specifically, a comprehensive analysis of individual polyphenols was performed on both fresh samples and the flours utilized in baked goods (Table 3). Regardless of by-products considered, the general trend showed that the concentration of most polyphenols was not strongly affected during the processing from fresh to flour, suggesting that the used conditions limited the degradation of antioxidants. In the case of grape pomace, although the decrease in catechin and epicatechin was not statistically significant, procyanidin B1 (a dimer of catechin and epicatechin) significantly increased. This increase could be explained by the fact that this polyphenol could be formed through two pathways: (i) polymerization/condensation of catechin and epicatechin [48] or (ii) depolymerization of high-molecular weight tannins [49]. Moreover, it seemed that epicatechin was more affected than catechin (49% vs. 21%; Table 3), likely because the former is less stable to heat treatment than the latter [41].

The trend of anthocyanins through the process on grape pomace was not uniform within the class; in fact, cyanidin-3-glucoside showed an increase in flour (107%) while the other exponents of the class did not undergo statistically significant changes. Processing of grape pomace did not significantly affect the quercetin aglycone content; however, individual quercetin derivatives responded differently. Quercetin-3-galactoside and quercetin-3-rhamnoside increased substantially in grape pomace flour—by 533% and 124%, respectively. The increase might be due to the depolymerization process, as previously discussed. In contrast, quercetin-3-glucoside and quercetin-3-glucuronide decreased by 32% and 38%, while quercetin-3,4′-diglucoside remained stable. These results suggest that mild drying at 40 °C combined with cryogenic grinding effectively preserved anthocyanin and quercetin compounds, likely by minimizing oxidation and thermal degradation.

In apple skins, total quercetin and its derivatives were higher than in grape pomace, but processing led to a decrease in all quercetin glycosides, while the aglycone increased by 11%. Specifically, quercetin-3-galactoside and quercetin-3-xyloside declined by 47% and 44%, and quercetin-3-glucoside and quercetin-3-rhamnoside by 36% and 30%, respectively. The greater tendency for the quercetin glycoside reduction observed in apple skins compared to grape pomace could be attributed to differences in processing conditions. While grape pomace was ground under cryogenic conditions, limiting oxidation and enzymatic activity, apple skins were ground at ambient temperatures, increasing their exposure to oxidative degradation. Despite the higher drying temperature (75 °C), a significant amount of quercetin was retained, suggesting moderate thermal stability under controlled conditions. These findings are consistent with previous studies indicating that quercetin glycosides are more susceptible to enzymatic hydrolysis and oxidation, while the aglycone form is relatively stable during thermal processing [2,50].

Chlorogenic acids in apple skins were unaffected by processing, consistent with He-ras-Ramírez et al. [47], who reported similar stability during air drying at 70 °C. Dihydro-chalcones, including phloretin, phloridzin, and their glycosides, showed only a minor decrease (9%), indicating greater thermal stability compared to flavonols. Their levels in Scilate apple skins aligned with prior data from André et al. [51], who reported dihydro-chalcones as comprising 3.5–29% of total apple polyphenols. These compounds, largely confined to the skin, are considered characteristic markers of apple-derived products [52].

Although less studied than other polyphenol classes, dihydrochalcones have recently gained attention for their health-promoting properties. In particular, phloretin and phloridzin have demonstrated antioxidant and anti-inflammatory effects, especially in the gut, where they may help regulate intestinal inflammations [53].

The observed differences in polyphenol content between grape and apple by-products, used in this study, may stem from matrix composition. The grape pomace included both skins and seeds, the latter being a well-documented source of polyphenols [54] whereas apple by-products consisted solely of skins. Moreover, the grape pomace used in this study underwent fermentation. The literature evidence suggests that fermentation can contribute to the release of bound polyphenols due to yeast enzymatic activity and the solvent effect of ethanol on seed cell walls, potentially increasing their bioavailability [52]. Thus, these two by-products are not entirely comparable.

The profile of the FAMEs was determined only in grape pomace flour as these do not occur in apple skins. This class of molecules is an important parameter to consider for the shelf life of foods because unsaturated fatty acids are easily oxidated. As reported in Supplementary Table S3, grape pomace flour has a high content of unsaturated fatty acids, namely MUFA and PUFA. The high grape pomace flour content of unsaturated fatty acids, around 86%, is in line with the profile for grape seeds in the literature [55]. It is worth noting that the total fat content of the evaluated grape pomace flour is 10.4 g/100 g.

### 3.3. Chemical and Technological Characterization of Baked Goods

The potential improvement in the antioxidant profile of the baked goods following the addition of grape pomace and apple skin was evaluated (Table 4).

Despite the relatively low addition levels of grape pomace (5%) and apple skin flours (3% in breadsticks and focaccia, 10% in cookies), all enriched baked goods exhibited a significant increase in antioxidant content compared to the unenriched (Table 4). Specifically, total polyphenols increased by 727%, 425%, and 300% when grape pomace and apple skin flours were added to the formulations of the breadsticks, focaccia, and cookies, respectively. This increase is consistent with the findings of Poiana et al. [56], who observed a similar rise in cookies enriched with grape pomace. While their study reported a 700% increase at a 25% enrichment level, our findings reveal a comparable improvement with only 10% incorporation (comprising 5% grape pomace and 5% apple skin flours), suggesting a more efficient preservation of bioactive compounds. Conversely, Giosuè et al. [14] observed a greater loss of anthocyanins in their fortified formulations, likely attributable to variations in drying parameters and grape cultivar. These comparisons underscore the critical role of optimized processing conditions in maximizing polyphenol retention. Notably, the substantial increase in anthocyanin content (ranging from 600% to 1718%) indicates that, despite their known thermal sensitivity, the baking process may not have significantly contributed to their degradation.

As regards the antioxidant capacity, breadsticks, which were baked at 210 °C, showed the highest increase in antioxidant capacity (1200%), likely due to the Maillard reaction, which generates antioxidant compounds [57]. In contrast, focaccia and cookies, baked at 190 and 170 °C, exhibited a lower increase (800 and 280%), possibly due to their higher moisture content (moisture content = 2.5 ± 0.2 vs. 35 ± 1% for breadsticks and focaccia, respectively) or lower temperature, which limits Maillard-derived antioxidant formation. These findings align with a previous study that demonstrated a direct correlation between baking temperature and antioxidant development [58]. The increase in anthocyanins and the antioxidant capacity observed in baked goods with grape pomace and apple skin flours, is in line with other authors who showed similar improvements with grape pomace flour in cookies and breads [56,59,60]. However, our data indicate an increase in antioxidant activity of up to 1200%, which is higher than values reported in previous studies, probably due to the specific phenolic composition of Lagrein pomace and apple skins [61,62]. In this context, Gómez-Brandón et al. [63] reported that phenolic compounds, particularly phenolic acids, anthocyanins, and other flavonoids, are likely the primary contributors to the antioxidant activity in grape pomace. These findings confirm that increasing the total polyphenol level in baked goods by adding grape pomace flour significantly increases their FRAP value. In contrast, some studies show a significant loss of antioxidant activity during cooking [64,65], suggesting that different factors, such as the food matrix, cooking time and temperature, and processing, can greatly influence the stability of bioactive compounds. These findings underscore the need to optimize baking parameters to maximize the functional contribution of these ingredients.

Notably, the increase in antioxidants in baked goods with added by-products was mainly due to grape pomace flour, which contributed significantly more (about 10 times) than apple flour (Table 4). However, focaccia with both grape pomace and apple skin was found to be more effective in promoting a healthy balance of gut microorganisms compared to focaccia with grape pomace flour alone [10]. Despite the grape pomace containing higher levels of polyphenols and anthocyanins than the apple skins, the presence of apple skins in the food formulation led to a more significant improvement in gut health. Specifically, the growth of beneficial microbiota species (e.g., Clostridium group IV) was enhanced by the presence of chlorogenic acids, which are found in apple skins but not in grape pomace [10].

Although the same amount of grape pomace (5%), was added to the baked goods, a significant discrepancy was observed in the antioxidant profile. These variations might be related to the different food formulations and processing conditions, such as the type and duration of leavening [65]. In this context, some authors demonstrated that yeast fermentation improves the bioavailability of polyphenols [66,67], explaining the greater increase in the antioxidant profile of the focaccia and breadsticks (biological leavening) compared to the cookies (chemical leavening).

Concerning the impact of baking on the stability of bioactive compounds, the results indicated that polyphenol retention ranged from about 90% to >100%, while anthocyanin retention ranged from about 90% to 161%, depending on the goods. In some cases, values above 100% suggest increased post-baking extractability or matrix effects. These values confirm that the applied processing conditions ensured substantial preservation of antioxidant compounds. Summary data are provided in Supplementary Table S5.

### 3.4. Texture, Color, and Sensory Attributes of Baked Goods

**Figure 2:**
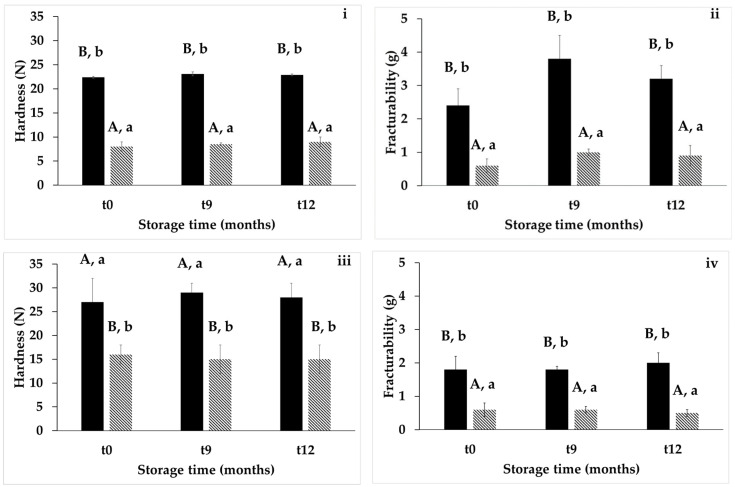
Hardness (**i**,**iii**) and fracturability (**ii**,**iv**) of unenriched (black bars) and enriched (white-black bars) breadsticks (**i**,**ii**), and cookies (**iii**,**iv**) with grape pomace and apple skin flours during storage (t0 = 2 h after baking, t9 and t12 = 9 and 12 months after baking). Different letters (lowercase letters refer to differences between unenriched and enriched sample at the same storage time; capital letters refer to same class of samples during storage) indicate significant differences (one-way ANOVA, Tukey HSD test, *p* ≤ 0.05).

From a technological standpoint, the addition of grape pomace and apple skin to baked goods impacted their textural and sensory properties. Firmness or hardness is particularly important in evaluating the essential texture characteristics analyzed for baked goods. It is a key indicator of freshness, making it a crucial factor in assessing baked goods [68,69]. Regarding the textural attributes (Figure 2) of breadsticks and cookies, the presence of by-products led to a decrease in their hardness (−64 and −41%, respectively) and fracturability (−75 and −67%, respectively), regardless of the storage time. Conversely, adding by-products increased the focaccia hardness (10.4 ± 2, 13 ± 2, 17 ± 5, 21 ± 4 N at 2 h, 1, 2, and 3 months after baking), compared with no addition (1.2 ± 0.4, 4.4 ± 0.2, 9.8 ± 0.8, and 12.0 ± 0.7 N at 2 h, 1, 2, and 3 months after baking). Similar trends were found by other authors following the addition of grape pomace in baked good formulations [70,71,72].

However, comparing the results with studies in the literature might be challenging due to the lack of texture profile analysis (TPA) parameters and varying measurement parameters, such as compression rates and levels. Additionally, some studies did not define the time after bread texture analysis was conducted or specify bread storage parameters, like temperature, making it difficult to compare results accurately. With a special mention of gluten-free products, different additives also influence the dough preparation and baking, affecting the bread’s properties.

Sensory attributes significantly influence consumer acceptability, with color being one of the main factors. Consumers place importance on the color of the crumb and crust in baked goods, such as bread, focaccia, and breadsticks. Fortifying baked goods with grape pomace and apple skin significantly impacted their internal color (Table 5).

Lightness decreased for all baked goods, while redness increased. Thus, this addition resulted in a decrease in brightness and an increase in dark red color in internal regions. The higher darkness of enriched baked goods might be related to the significant presence of polyphenols in grape pomace, particularly anthocyanins, which display a purple–red color at baking pH. The increased yellowness value in cookies could be related to intensified Maillard and caramelization reactions, supported by the higher sugar content and baking conditions. Indeed, the formation of Maillard-derived pigments is favored by low water activity, sour dough (acidic dough), and the presence of sugars. This is consistent with Jannatti et al. [73], who reported increased yellowness in apple-enriched baked goods. The instrumental findings correlate well with sensory data, where darker samples received lower color acceptability scores.

### 3.5. Consumer Acceptance and Perception of Sustainability

**Figure 3:**
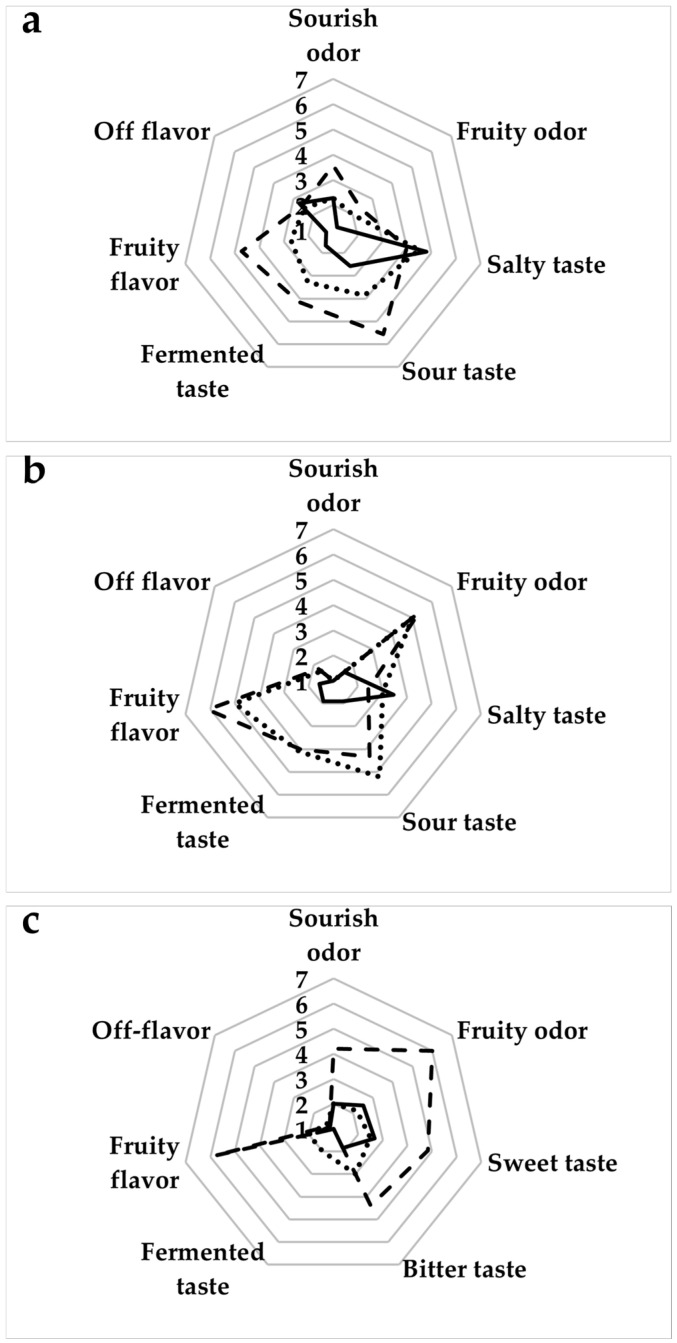
Sensory evaluation of the breadsticks (**a**), focaccia (**b**), and cookies (**c**) prepared without by-products (unenriched sample; solid line), with grape pomace flour (dotted line), with grape pomace and apple skin flour (dashed line).

Moreover, the baked goods were sensory evaluated using a nine-point hedonic scale. The mean ratings for various attributes, including odor, flavor, and taste, are presented in Figure 3. Adding by-products enhanced the overall intensity of fragrance, particularly in wine and fruit notes, indicating that already a 5% fortification was able to improve the flavor and odor of the baked goods. Additionally, the use of grape pomace alone or combined with apple skin led to more pronounced sour flavor. This result agreed with the study by Bucalossi et al. [74], who reported that the presence of phenolic compounds from grapes in vegetable-based food systems increased sourness perception. Off-flavors were significantly higher in focaccia when grape pomace and apple skin were added. For cookies, the inclusion of apple skin increased both sweetness and bitterness.

Incorporating grape pomace alone or combined with apple skin in breadsticks was well-received, with no significant differences from the unenriched sample. On the other hand, the consumers appreciated the focaccia with the grape pomace flour alone less than the same product with grape pomace and apple skin, resulting in no significant difference from the unenriched sample. The decrease in consumers’ acceptance of cereal-based products with the addition of grape pomace flour has already been reported by Rosales Soto et al. [75]. Based on our results, the presence of apple skin flour slightly improved the acceptability of baked goods with added grape pomace (Table 6).

Regarding the breadsticks, the samples generally met the sensory criteria, with the unenriched sample excelling in color and flavor, while the sample with grape pomace and apple skin stood out for mouthfeel (Table 6). For focaccia, the unenriched sample performed best across all attributes. Enriched samples had mixed approval for all attributes evaluated. The color ratings for all samples were similar to those observed by other researchers [75,76], with lower scores for grape pomace addition, likely due to the darker color of the products than the control. This darker color suggests that grape pomace could be a natural coloring agent in baked goods, contributing to their characteristic browned look [63,76]. Finally, the acceptance questionnaire (Table 7) showed that most consumers recognize the importance of food for their health, with over 70% expressing a willingness to buy products enhanced with food industry by-products to improve nutritional content. Nevertheless, no consensus existed on whether enriched foods could be inherently healthier, compared to unenriched foods, or would help meet daily dietary recommendations. Only 27% would choose a product with reduced taste even if it were enriched. Notably, 80% of consumers are open to purchasing products enriched with food industry by-products to enhance nutrition and support eco-circulation. Significant distinctions arose primarily among the focaccia samples concerning consumer preferences for breadsticks and focaccia (Table 7). The control sample garnered the highest liking score and demonstrated statistical similarity to the sample enriched with grape pomace and apple skin. The latter, however, achieved a liking score comparable to the sample with only grape pomace. Although no significant differences in preference were shown among the breadsticks, the sample with the highest score was the one enriched with grape pomace and apple skins. The sensory profiles of the enriched samples revealed that while grape pomace flour alone reduced color and flavor scores due to its intense pigmentation and slight astringency, its combined use with apple peel flour improved overall acceptability. This suggests that formulation synergy can offset potential sensory drawbacks, particularly in breadsticks and focaccia. The results are consistent with increasing consumer interest in sustainability and functional foods, especially when sensory properties remain acceptable [75,76]. These findings support the potential of upcycled ingredients in gluten-free product innovation.

## 4. Conclusions

This study demonstrated the feasibility of adding grape pomace and apple skin by-products as functional ingredients in gluten-free baked goods. Mild drying and optimized grinding preserved key polyphenols and anthocyanins, which remained bioavailable even after baking. The incorporation of these flours significantly improved the antioxidant capacity of breadsticks, focaccia, and cookies without compromising consumer acceptability.

Notably, combining grape and apple by-products not only enhanced nutritional value but also contributed to a more favorable sensory profile compared to grape pomace alone. This suggests that a tailored formulation can mitigate the typical limitations of polyphenol-rich matrices in gluten-free products.

Overall, the upcycling approach supports a circular food system and offers a viable solution to improve the health profile of gluten-free bakery items. Future studies should explore the bio-accessibility of these compounds. However, ensuring standardization in raw material processing and optimizing storage conditions remain the key challenges for a large-scale implementation.

## Figures and Tables

**Table 1:** Amount of grape pomace and apple skin flours used to produce baked goods.

Food Systems	Grape Pomace Flour	Apple Skin Flour	Other Ingredients
Unenriched breadsticks	-	-	See Section 2.4.1
Enriched breadsticks	5%	3%	
Unenriched focaccia	-	-	See Section 2.4.2
Enriched focaccia	5%	3%	
Unenriched cookies	-	-	See Section 2.4.3
Enriched cookies	5%	10%	

**Table 2:** Total polyphenols, total anthocyanins, and antioxidant capacity of fresh and dried grape pomace and apple skins, and of the resulting flours.

		Fresh	Dried	Flour
Grape pomace	Total polyphenols	4045.3 ± 105.5 ^b^	3661.1 ± 127.3 ^a^	4369.9 ± 173.3 ^b^
	Total anthocyanins	389.6 ± 8.0 ^c^	262.4 ± 9.5 ^a^	335.2 ± 15.8 ^b^
	Antioxidant capacity	80.4 ± 4.1 ^b^	71.2 ± 4.0 ^a^	76.9 ± 3.3 ^ab^
Apple skin	Total polyphenols	683.6 ± 52.8 ^a^	587.9 ± 24.0 ^a^	556.3 ± 4.2 ^a^
	Total anthocyanins	32.1 ± 2.3 ^b^	21.0 ± 1.2 ^a^	20.5 ± 1.0 ^a^
	Antioxidant capacity	10.6 ± 0.1 ^c^	9.2 ± 0.0 ^b^	7.9 ± 0.0 ^a^

Total polyphenols (Folin–Ciocalteu), total anthocyanins, and antioxidant capacity (FRAP) are expressed as mg_GAE_/100 g DW, mg_Cya-3-glu eq_/100 g DW, and mmol Fe^2+^/100 g DW, respectively. Different letters in the same row indicate a statistical difference between samples (one-way ANOVA, Tukey HSD, *p* < 0.05). Grape pomace flour: flour from cryogenic grinding of dried grape pomace; apple skin flour: flour from lab-scale grinding (ZM200 Retsch mill) of dried apple skin.

**Table 3:** Comparison of single polyphenols on fresh by-products and related flours.

Compound Class	Compound	Fresh Grape Pomace	Grape Pomace Flour	Fresh Apple Skin	Apple Skin Flour
Flavan-3-ols	Catechin	64.9 ± 0.2	51.1 ± 4.1 ^ns^	2.1 ± 0.1	1.4 ± 0.0 **
	Epicatechin	46.8 ± 1.2	23.8 ± 9.1 ^ns^	26.8 ± 1.0	21.3 ± 0.0 *
	Procyanidin B1	19.3 ± 2.1	47.2 ± 4.3 *	3.4 ± 0.2	2.6 ± 0.0 *
	Procyanidin B2	17.3 ± 0.1	19.0 ± 3.9 ^ns^	29.7 ± 1.1	30.4± 0.1 *
	Procyanidin C1	13.4 ± 0.7	14.3 ± 1.0 ^ns^	26.5 ± 1.0	19.5 ± 0.2 **
Anthocyanins	Cyanidin-3-glucoside	1.4 ± 0.1	2.9 ± 0.3 *	n.d.	n.d.
	Cyanidin-3-galactoside	n.d.	n.d.	19.6 ± 0.8	10.2 ± 0.3 **
	Cyanidin-3-arabinoside	n.d.	n.d.	1.0 ± 0.0	0.4 ± 0.1 *
	Petunidin-3-glucoside	n.d.	11.3	-	-
	Delphinidin-3-glucoside	7.2 ± 0.0	6.7 ± 0.7 ^ns^	-	-
Flavonols	Quercetin	12.5 ± 4.8	9.7 ± 2.7 ns	8.3 ± 0.0	9.3 ± 0.2 *
	Quercetin-3-arabinoside	n.d.	n.d.	13.2 ± 0.2	7.9 ± 0.3 **
	Quercetin-3-galactoside	1.1 ± 0.0	6.9 ± 0.8 **	51.4 ± 0.2	27.4 ± 2.1 **
	Quercetin-3-rhamnoside	0.7 ± 0.0	1.5 ± 0.2 *	11.2 ± 0.2	7.8 ± 0.2 **
	Quercetin-3,4-diglucoside	1.3 ± 0.0	1.2 ± 0.1 ^ns^	-	-
	Quercetin-3-glucoside	3.5 ± 0.1	2.4 ± 0.4 *	7.8 ± 0.3	5.0 ± 0.5 *
	Quercetin-3-glucuronide	8.4 ± 0.0	5.2 ± 0.2 ***	-	-
	Quercetin-3-xyloside	n.d.	n.d.	26.7 ± 0.5	15.0 ± 0.5 **
	Rutin	n.d.	n.d.	3.3 ± 0.1	2.1 ± 0.3 *
	Isorhamnetin-3-glucoside	0.6 ± 0.0	2.0 ± 0.3 *	-	-
	Kaempferol	n.d.	1.4 ± 0.2	-	-
	Kaempferol-3-glucoside	n.d.	2.0 ± 0.3	-	-
	Kaempferol-3-glucuronide	0.2 ± 0.0	2.7 ± 0.4 *	-	-
	Kaempferol-3-rutenoside	0.6 ± 0.0	n.d.	-	-
	Myricetin	5.2 ± 0.2	8.1 ± 1.5 ^ns^	-	-
	Myricetin-3-glucoside	2.9 ± 0.0	2.5 ± 0.5 ^ns^	-	-
Hydroxycinnamic acids	Caftaric acid	1.7 ± 0.3	2.9 ± 0.2 *	-	-
Sum of Chlorogenic acids		-	-	2.7 ± 0.1	2.6 ± 0.0 ^ns^
Sum of Dihydrochalcones		-	-	27.4 ± 0.3	25.0 ± 0.7 *

All compounds are expressed as mg/100 g DW. Asterisks in the same row for the same by-products indicate a statistical difference between samples (*t*-Test, * *p* ≤ 0.05, ** *p* ≤ 0.01, *** *p* ≤ 0.001). The following analytes were not detected, so they are not listed in the table (see Supplementary Tables S1 and S2): astilbin, epigallocatechin, isorhamnetin, isorhamnetin-3-glucoside, malvin, malvidin-3-glucoside, petunidin, prunin, taxifollin. n.d. = not detected; ns = no statistical difference; “-” = not analyzed.

**Table 4:** Total polyphenols, total anthocyanins, and antioxidant capacity in unenriched and enriched by-products (grape pomace and apple skin) baked goods.

		Total Polyphenols	Total Anthocyanin	Antioxidant Capacity
Breadsticks	unenriched	26.1 ± 0.6	1.6 ± 0.1	0.3 ± 0.0
	enriched	215.3 ± 8.5 ***	15.7 ± 0.3 ***	4.1 ± 0.0 ***
Focaccia	unenriched	59.2 ± 3.9	1.1 ± 0.1	0.6 ± 0.3
	enriched	310.2 ± 3.0 ***	20.0 ± 0.7 ***	5.4 ± 0.1 ***
Cookies	unenriched	87.3 ± 22.3	4.1 ± 0.0	1.3 ± 0.0
	enriched	349.4 ± 7.2 ***	29.5 ± 1.0 ***	4.8 ± 0.2 ***

Total polyphenols (Folin–Ciocalteu), anthocyanin content, and antioxidant capacity (FRAP) are expressed as mg_GAE_/100 g DW, mg_Cya-3-glu_/100 g DW, and mmol Fe^2+^/100 g DW, respectively. The asterisks indicate significant differences between the means of the unenriched and enriched samples of each class (*t*-Test; *** *p* ≤ 0.001).

**Table 5:** Internal color of unenriched and enriched by-products (grape pomace and apple skin) baked goods.

		Luminosity	Redness	Yellowness
Breadsticks	unenriched	83.0 ± 0.9	−3.0 ± 0.3	19.9 ± 0.6
	enriched	30.3 ± 2.3 ***	6.5 ± 0.2 ***	8.1 ± 0.3 ***
Focaccia	unenriched	70.2 ± 1.3	3.0 ± 0.2	27.6 ±1.7
	enriched	31.9 ± 1.7 ***	7.4 ± 0.3 ***	6.3 ± 0.4 ***
Cookies	unenriched	69.9 ± 0.7	3.2 ± 0.2	8.6 ± 0.3
	enriched	32.4 ± 2.0 ***	9.6 ± 0.8 ***	2.38 ± 2.0 ***

The asterisks indicate significant differences between the means of the control unenriched and enriched samples of each class (*t*-Test; *** *p* ≤ 0.001).

**Table 6:** Mean liking scores (%) and appropriateness of sensory attributes (%) for baked goods in three different conditions (unenriched sample; with grape pomace; with grape pomace and apple skin).

Sample		Unenriched	Grape Pomace Flour	Grape Pomace + Apple Skin Flours
Breadsticks		71.7 ± 2.1 ^a^	72.3 ± 1.4 ^a^	74.1 ± 1.6 ^a^
Focaccia		63.0 ± 1.8 ^b^	55.7 ± 1.6 ^a^	58.9 ± 1.9 ^ab^
Breadsticks	Color	93.8 ± 1.5 ^c^	70.4 ± 1.7 ^a^	79.4 ± 1.3 ^b^
	Flavor	91.2 ± 1.4 ^b^	84.7 ± 1.5 ^a^	85.2 ± 1.1 ^a^
	Mouthfeel	87.5 ± 1.6 ^b^	84.1 ± 1.4 ^a^	91.6 ± 1.6 ^c^
Focaccia	Color	99.0 ± 1.0 ^b^	56.2 ± 1.6 ^a^	56.4 ± 1.7 ^a^
	Flavor	87.4 ± 1.4 ^b^	68.4 ± 1.1 ^ab^	63.3 ± 1.4 ^a^
	Mouthfeel	87.3 ± 1.7 ^c^	65.9 ± 1.5 ^a^	83.1 ± 1.2 ^b^

Different letters in the same row indicate significant differences (one-way ANOVA, Tukey HSD test, *p* ≤ 0.05).

**Table 7:** Consumer acceptance toward enriched food products, sustainability, eco-circularity, and recovery of by-products from the food industry. Data reported as frequency distribution (%, *n* = 75).

Item Pool	Strongly Disagree	Disagree	Neutral	Agree	Strongly Agree
1. Food plays an important role in my personal health	-	-	-	15	85
2. I think I would buy a product enriched with food industry by-products, such as grapes and apples, which are rich in antioxidants and/or fiber, in order to improve their nutritional characteristics	-	5	16	45	33
3. Functional foods are likely to have a beneficial impact on my personal health	-	3	19	45	33
4. Functional foods are a convenient way of meeting recommended daily intakes, which I would never meet with my conventional diet	7	31	27	24	12
5. I do believe that enriched foods are considered healthier than traditional ones	12	33	40	12	3
6. Functional foods are acceptable for me, even if they taste worse than the conventional alternative foods	4	31	39	21	5
7. According to my personal opinion, I would purchase a product enriched with food industry by-products to enhance and promote the concept of reusing such resources in the production of new foods	3	7	15	40	36

## Data Availability

Data is contained within the article and Supplementary Materials.

## Supplementary Materials

The following supporting information can be downloaded at: https://www.mdpi.com/article/10.3390/antiox14070798/s1. Supplementary Table S1: Mass spectrometric parameters applied for each analyte for determination of single polyphenols of grape pomace. Supplementary Table S2: Mass spectrometric parameters applied for each analyte for determination of single polyphenols of apple skins. Supplementary Table S3: Total fat content, expressed in g/100 g, and FAME profile, expressed as a relative percentage (%), of the grape pomace flour. For FAME, besides single FAMEs, the classes saturated fatty acids (SAFAs), monounsaturated fatty acids (MUFA) and polyunsaturated fatty acids (PUFA) are reported. Supplementary Table S4: Particle size distribution on grape pomace flour sample: comparison between laboratory miller vs. industrial miller. Supplementary Table S5: Impact of baking on the stability of bioactive compounds after baking, on baked goods, compared with theoretical amount added with each by-product flour.

## Abbreviations

The following abbreviations are used in this manuscript:

HPLC-MS	High performance liquid chromatography–mass spectrometry
UHPLC	Ultra-high performance liquid chromatography
H-ESI	Heated–electrospray ionization
LD	Linear dichroism
SAFA	Saturated fatty acid
MUFA	Mono-unsaturated fatty acid
PUFA	Poly-unsaturated fatty acid
FRAP	Ferric-reducing antioxidant potential
TPC	Total polyphenol content
AC	Anthocyanin content
FAME	Fatty acid methyl esters
GC-FID	Gas chromatography–flame ionization detector
DW	Dry weight
ARB	Arbitrary unit
