# Supplementary material for: Upcycling of By-Products from Autochthonous Red Grapes and Commercial Apples as Ingredients in Baked Goods: A Comprehensive Study from Processing to Consumer Consumption
Source: Antioxidants (Basel). 2025 Jun 27;14(7):798. doi: 10.3390/antiox14070798 (PMC12291893; doi:10.3390/antiox14070798)
Supplement: Supplementary file 1 [file antioxidants-14-00798-s001.zip › antioxidants-3699938-supplementary.pdf]

Supplementary Material

Upcycling of By-Products from Autochthonous Red Grapes and Commercial Apples as Ingredients in Baked Goods: A Comprehensive Study from Processing to Consumer Consumption

Gaetano Cardone [1,†], Martina Magni [2,†], Veronica Marin [1], Andrea Pichler [2], Daniele Zatelli [3], Peter Robatscher [2,*], Ombretta Polenghi [1], Virna Lucia Cerne [1], Michael Oberhuber [2] and Silvano Ciani [1,*]

[1] Dr. Schär Research & Development Department, 34139 Trieste, Italy; gaetano.cardone@drschaer.com (G.C.); veronica.marin@drschaer.com (V.M.); ombretta.polenghi@drschaer.com (O.P.); virna.cerne@drschaer.com (V.L.C.)

[2] Laboratory of Flavours and Metabolites, Laimburg Research Centre, Laimburg 6, 39040 Auer-Ora, Italy; andrea.pichler@laimburg.it (A.P.); michael.oberhuber@laimburg.it (M.O.)

[3] VOG Products, Soc. Agricola Coop, 39055 Laives, Italy; daniele.zatelli@vog-products.it

* Correspondence: peter.robatscher@laimburg.it (P.R.); silvano.ciani@drschaer.com (S.C.)

† These authors contributed equally to this work.

Supplementary Table S1. Mass spectrometric parameters applied for each analyte for determination of singles polyphenols of grape pomace.

Compound	Retention Time (min)	Retention Time Window (min)	Polarity	Precursor (m/z)	Product (m/z)	Collision Energy (V)	RF Lens (V)
Catechin-2,3,4-$^{13}C_3$ (IS)	5.5	4	Positive	294.2	124.1	15.0	55
				294.2	140.1	17.0	55
Epigallocatechin	5.5	4	Positive	307.1	139.0	17.2	53
				307.1	163.0	22.0	53
Procyanidin B1	5.5	4	Positive	579.2	291.1	14	78
				579.2	427.1	15	78
Caftaric acid	5.8	4	Negative	311.0	149.0	10.2	49
				311.0	216.8	11.6	49
Catechin	5.9	4	Positive	291.1	123.1	15.9	52
				291.1	139.1	17.3	52
Delphinidin-3-glucoside	6.0	4	Positive	465.2	229.1	52.0	73
				465.2	303.1	22.6	73
Epicatechin	6.0	4	Positive	291.1	123.1	15.9	52
				291.1	139.0	17.2	52
Malvin	6.0	4	Positive	655.6	331.1	30.7	95
				655.6	493.2	18.7	95
Cyanidin-3-galactoside	6.2	4	Positive	449.3	213.1	53.0	72
				449.3	287.1	23.0	72
Gallocatechin	6.5	4	Positive	307.1	139.0	17.2	53
				307.1	163.0	22.0	53
Cyanidin-3-glucoside	6.5	4	Positive	449.3	213.1	51.0	72
				449.3	287.1	22.0	72
Petunidin-3-glucoside	6.7	4	Positive	479.5	302.1	40.2	72
				479.5	317.1	21.4	72
Cyanidin-3-arabinoside	6.8	4	Positive	419.2	213.1	48.0	69
				419.2	287.1	20.0	69
Procyanidin B2	7.0	4	Positive	579.2	291.1	14.0	78
				579.2	427.1	14.6	78
Malvidin-3-glucoside	7.6	4	Positive	493.4	287.0	30.0	95
				493.4	331.3	15.0	95
Myricetin-3-glucoside	8.2	4	Positive	481.1	273.0	37.9	55
				481.1	319.1	14.0	55

(continue on the next page)

Petunidin	8.2	4	Positive	317.2	155.1	15.0	73
				317.2	177.0	21.0	73
Procianidin_C1	8.5	4	Positive	867.2	409.1	29.1	97
				867.2	579.1	14.4	97
Quercetin-3,4-diglucoside	8.8	4	Positive	627.1	303.0	29.5	69
				627.1	465.1	11.9	69
Quercetin-3-galactoside	9.0	4	Positive	465.1	303.0	15.0	52
Quercetin-3-glucoside	9.2	4	Positive	465.1	303.0	14.1	54
Quercetin-3-glucuronide	9.2	4	Positive	479.4	303.1	16.8	63
Astilbin	9.5	4	Positive	451.2	305.1	10.2	40
				451.2	410.7	10.2	40
Taxifollin	9.8	4	Positive	305.1	153.1	16.1	55
				305.1	259.0	13.6	55
Kaempferol-3-glucuronide	10.1	4	Positive	463.2	287.0	16.7	62
Isorhamnetin-3-glucoside	10.2	4	Positive	479.2	302.0	38.2	56
				479.2	317.1	13.7	56
Myricetin	10.3	4	Positive	319.1	153.0	31.4	88
				319.1	217.1	31.1	88
Kaempferol	10.5	4	Positive	287.1	153.1	33.0	97
				287.1	165.0	27.6	97
Kaempferol-3-glucoside	10.5	4	Positive	449.1	287.0	14.2	51
Quercetin-3-arabinoside	10.7	4	Positive	435.1	303.0	14.0	49
Isorhamnetin	10.8	4	Positive	317.2	275.8	10.2	81
				317.2	302.0	24.1	81
Kaempferol-3-rutenoside	10.9	4	Positive	595.2	287.1	13.0	60
				595.2	449.1	13.0	60
Isorhamnetin-3-rutenoside	10.9	4	Positive	625.2	317.1	20.6	64
				625.2	479.1	11.2	64
Quercetin-3-rhamnoside	11.0	4	Positive	449.1	303.0	10.2	45
Quercetin	13.0	4	Positive	303.0	153.1	33.5	90
				303.0	229.0	29.1	90

Supplementary Table S2. Mass spectrometric parameters applied for each analyte for determination of singles polyphenols of apple skins.

Compound	Retention Time (min)	Retention Time Window (min)	Polarity	Precursor (m/z)	Product (m/z)	Collision Energy (V)	RF Lens (V)
Neochlorogenic acid	1.7	2	Positive	355.2	163.1	10.2	46
Procyanidin B1	2.2	2	Positive	579.2	291.1	10.2	89
Catechin	2.6	2	Positive	291.2	123.1	15.1	60
				291.2	139.0	15.9	60
Chlorogenic acids	3.5	2	Positive	355.2	163.1	12.0	57
Procyanidin B2	3.7	2	Positive	579.2	299.0	25.9	88
Epicatechin	4.4	2	Positive	291.1	123.1	15.6	60
				291.1	139.1	16.1	60
Cyanidin-3-galactoside	4.4	2	Positive	449.2	287.0	22.0	75
Cyanidin-3-glucoside	4.9	2	Positive	449.2	287.1	22.0	75
Cyanidin-3-arabinoside	5.2	2	Positive	419.2	287.1	21.0	71
Procyanidin C1	5.2	2	Positive	867.2	407.1	27.8	102
				867.2	577.1	14.4	102
				867.2	579.1	14.3	102
Phloretin-2-xyloglucoside	10.4	2	Positive	569.0	107.1	18.5	55
				569.0	275.0	18.5	55
Malvidin-3-glucoside	6.8	2	Positive	493.2	287.0	28.0	81
				493.2	331.1	20.9	81
Quercetin-3-galactoside	7.5	3	Positive	465.1	303.0	13.0	61
Quercetin-3-glucoside	7.9	3	Positive	465.0	303.0	13.0	61
Rutin	7.8	2	Positive	611.2	303.1	20.5	70
Quercetin-3-xyloside/arabinoside	8.5	3	Positive	435.2	303.0	11.5	59
Quercetin-3-rhamnoside	9.3	3	Positive	449.2	303.0	10.2	53
Prunin	9.2	2	Positive	435.2	273.1	13.7	61
Phloretin	12.0	3	Positive	275.2	107.1	18.5	55
Phloridzin	10.7	3	Positive	437.2	275.1	10.2	60
				437.2	317.1	10.2	60
Quercetin	12.0	3	Positive	303.3	153.1	32.0	93
				303.3	229.0	28.0	93

Supplementary Table S3. Total fat content, expressed in g/100 g, and FAME profile, expressed as a relative percentage (%), of the grape pomace flour. For FAME, besides single FAMEs, the classes saturated fatty acids (SAFAs), monounsaturated fatty acids (MUFA) and polyunsaturated fatty acids (PUFA) are reported.

**GRAPE POMACE FLOUR**	
TOTAL FAT CONTENT	*(g/100 g)*
	10.4
FATTY ACIDS	(%)
C 14	0.1
C 15	0.0
C 16	8.1
C 16:1	0.3
C 17	0.1
C 17:1	0.0
C 18	4.4
tC-18:1	0.0
C 18:1	17.8
tC-18:2	0.0
C 18:2	66.9
C 20	n.d.
C 20:1	0.2
C 18:3n3	0.8
C 20:2	0.0
C 22	0.1
C 22:1	0.1
C 24	0.0
C 22:6	0.0
**SAFA**	**12.8**
**MUFA**	**18.4**
**PUFA**	**67.7**
Unknown	1.1
Total (SAFA+MUFA+PUFA)	98.9

Supplementary Table S4. Particle size distribution (%) on grape pomace flour sample: comparison between laboratory miller (Retsch® MM 400 Mill; Verder Scientific GmbH & Co. K.G., Golling, Austria) *vs* industrial miller (CRIO-Contraplex 250 CWII, Hosokawa Alpine AG, Augsburg, Germany).

	**Particle size distribution (%)**			
Dimensional classes	**< 150 µm**	**150 – 250 µm**	**250 – 355 µm**	**> 355 µm**
Laboratory mill	26.7 ± 0.3	20.4 ± 0.3	12.3 ± 0.1	40.6 ± 0.1
Industrial mill	55.8 ± 1.7	16.4 ± 0.9	10.4 ± 2.1	17.4 ± 1.4

Supplementary Table S5. Impact of baking on the stability of bioactive compounds after baking, on baked goods, compared with theoretical amount added with each by-product flour.

			**Total Polyphenols**			**Total Anthocyanins**		
**Baked goods**	**Grape Pomace Flour (%)**	**Apple Skin Flour (%)**	**Theoretical (mg/100 g)**	**Measured (mg/100 g)**	**Retention (%)**	**Theoretical (mg/100 g)**	**Measured (mg/100 g)**	**Retention (%)**
Breadsticks	5	3	235.2	215.0	91 %	17.4	15.8	91 %
Focaccia	5	3	235.2	310.0	132 %	17.4	20.0	115 %
Cookies	5	10	274.1	349.0	127 %	18.0	29.0	161 %

Note: Theoretical values were calculated based on the content of each antioxidant in grape pomace flour (4,370 mg_{GAE}/100 g for total polyphenols, 335 $mg_{Cya\text{-}3\text{-}glu\ eq}$/100 g for total anthocyanins) and apple skin flour (556 mg_{GAE}/100 g, 21 $mg_{Cya\text{-}3\text{-}glu\ eq}$/100 g respectively), multiplied by the percentage of inclusion in the formulation. All values refer to dry weight (DW).
